# Vector compositions change across forested to deforested ecotones in emerging areas of zoonotic malaria transmission in Malaysia

**DOI:** 10.1038/s41598-019-49842-2

**Published:** 2019-09-16

**Authors:** Frances M. Hawkes, Benny O. Manin, Amanda Cooper, Sylvia Daim, Homathevi R., Jenarun Jelip, Tanrang Husin, Tock H. Chua

**Affiliations:** 1 Natural Resources Institute, University of Greenwich at Medway, Chatham Maritime, Kent, ME4 4TB UK; 2 Universiti Malaysia Sabah, Kota Kinabalu, Sabah 88400 Malaysia; 3 Royal Botanic Gardens Kew, Richmond, Surrey, TW9 3AE UK; 4 Disease Control Division, Ministry of Health, Federal Government Administration Centre, Putrajaya, Malaysia; 5 Division of Public Health, Sabah Department of Health, Kota Kinabalu, Sabah Malaysia

**Keywords:** Entomology, Biodiversity

## Abstract

In lowland areas of Malaysia, *Plasmodium knowlesi* infection is associated with land use change and high proportions of the vector *Anopheles balabacensis*. We conducted a 15-month study in two Malaysian villages to determine the effect of habitat on vector populations in understudied high-altitude, high-incidence districts. *Anopheles* mosquitoes were sampled in human settlements, plantations and forest edges, and screened for *Plasmodium* species by PCR. We report the first *An. donaldi* positive for *P. knowlesi*. This potential vector was associated with habitat fragmentation measured as disturbed forest edge:area ratio, while *An. balabacensis* was not, indicating fragmented land use could favour *An. donaldi*. Anopheline species richness and diversity decreased from forest edge, to plantation, to human settlement. Greater numbers of *An. balabacensis* and *An. donaldi* were found in forest edges compared to human settlements, suggesting exposure to vectors and associated zoonoses may be greater for people entering this habitat.

## Introduction

In South East Asia, the long-tailed macaque (*Macaca fascicularis*) is the main reservoir of at least five simian malarias, namely, *Plasmodium coatneyi*, *P*. *inui*, *P*. *fieldi*, *P*. *cynomolgi* and *P*. *knowles*i^1,2^. Of these, *P. knowlesi* now routinely infects humans. This zoonotic malaria is particularly problematic in Sabah, Malaysian Borneo, where it is currently the prevalent cause of clinical malaria^3,4^. Another of these species, *P*. *cynomolgi*, previously only shown experimentally to infect humans, has been reported in naturally acquired human cases, first as a single case in peninsular Malaysia^5^ and, more recently, in five cases in Kapit district of Sarawak, Malaysian Borneo^6^. Thus, as previously predicted^7^, simian malaria parasites are now contributing to clinical malaria in humans, derailing regional efforts to eradicate the disease. This is of particular public health importance in Malaysia, which aims to eradicate malaria by 2020.

In 2016, the World Health Organization Malaria Policy Advisory Committee reported that *P*. *knowlesi* was the predominant malaria species in Malaysia, comprising 69% of cases^8^. In Sabah state, this species was responsible for the majority of malaria cases, with 815 and 996 cases reported in 2012 and 2013, respectively^9^. The most recent study showed that the proportion of *P. knowlesi* among indigenous malaria cases has continued to increase, rising from 80% in 2015 to 88% in 2016, and then again in 2017 to 98% of all malaria admissions in the state^10^.

Recent findings have demonstrated a clear link between land use change and *P*. *knowlesi* incidence in this region, which strongly supports the idea that an epidemiological change is taking place^11^. A high *P*. *knowlesi* incidence was recorded in Sabah, where land use changes have occurred, such as opening of forest areas for commercial plantations and logging, with the proportion of *P*. *knowlesi* of all malaria cases in Sabah for 2014-2018 being 0.66, 0.71, 0.69, 0.88 and 0.89 respectively (2584/3925, 1640/2323, 1600/2318, 3614/4114 and 4131/4630 respectively)^12^. The large-scale land use changes observed in the region are likely to impact the ecology, behavior and transmission potential of *Anopheles* mosquitoes as vectors of zoonotic malaria^13^. Yet data on the degree of contact between vectors and human hosts and how this differs according to land use type in the context of rapid anthropogenic environmental change are not well understood^14^. Understanding current human infection risk from simian malaria species and anticipating possible future zoonotic spillover of vector-borne disease calls for a more comprehensive examination of local anopheline population ecology, as well as surveillance of their *Plasmodium* infection status.

We hypothesized that vector composition would change across an ecotone of increasing forest disturbance. We therefore set out to characterize the abundance, diversity and transmission potential of *Anopheles* species, especially known *Plasmodium* vectors, biting humans in three habitat types representative of predominant land uses in rural Sabah, namely human settlements, plantations and forest edges. To do this, we carried out a comparative study focusing on *Anopheles* mosquitoes in two high altitude areas in understudied regions of Sabah that nonetheless experience a high burden of human infection with *P. knowlesi* (Supplementary Table S1) and are undergoing environmental change. Our results are discussed with reference to known *Anopheles* species compositions in areas with well-documented vector profiles and the wider challenge of predicting changes in vector populations that may arise from deforestation and agricultural development.

## Methods

### Study sites

The study was carried out in Ranau and Keningau (Table 1), two inland districts situated at higher altitudes than Kudat where most previous studies on *P. knowlesi* in Sabah have been conducted. Both areas reported human cases in the year prior to data collection^15^.

**Table 1:** Summary of study site details for Ranau and Keningau districts.

District	Area (km^2^)	Elevation (meter above sea level)	Population (2010)	Reported *P. knowlesi* cases		Study village (coordinates)
				2013	2014	
Ranau	3,555.51	236 m	94,092	172	166	Paus (5°41′N, 116°47′E)
Keningau	3,532.82	490 m	200,985	85	169	Keritan Ulu (5°19′N, 116°2′E)

**Figure 1:**
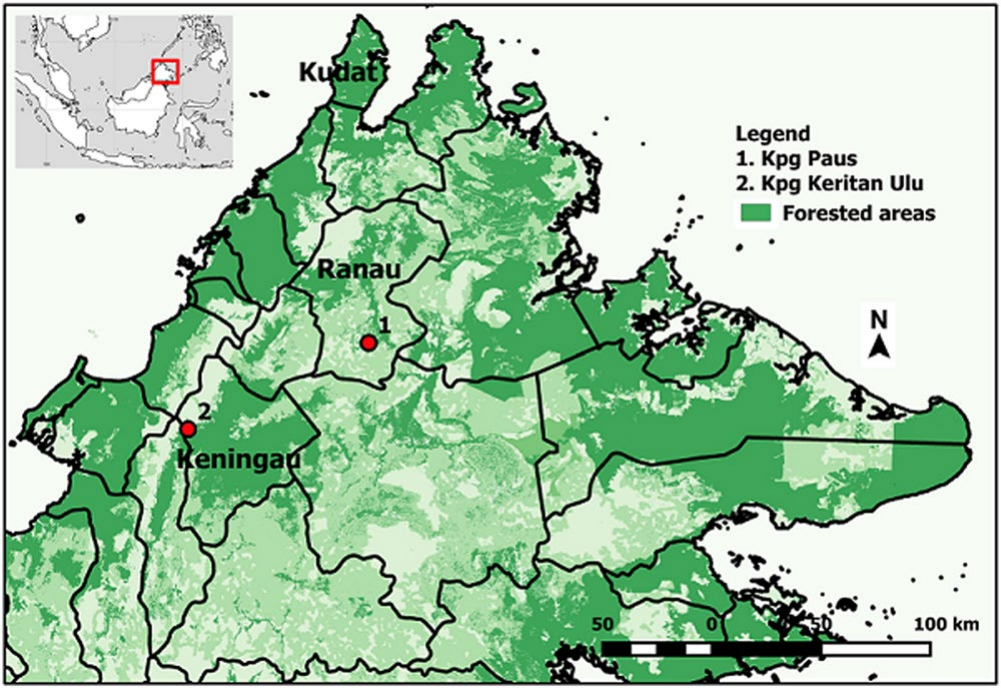
Location of study sites in Ranau and Keningau. 1 = Kampung Paus, 2 = Kampung Keritan Ulu.

Ranau district is in the west coast division of Sabah, about 108 km east of Kota Kinabalau. The northern part of Ranau is bounded by the Crocker Range and the Pinousok summit. Sampling was undertaken in Kampung Paus, which is approximately 80 km from Ranau town (Fig. 1). Keningau district is situated in a valley bordered by the Crocker Range to the west and the Trus Madi Range to the east and south. Sampling here was performed at Kampung Keritan Ulu, approximately 32 km from Keningau town. Both villages have and are surrounded by abundant and varied potential breeding sites for *Anopheles* species, including shaded and sunlit forest ground pools, irrigated rice fields, rock pools adjacent to rivers, stumps in bamboo stands, springs, water-filled animal hoof prints and ephemeral puddles.

### Collection of *Anopheles* using human landing catches

At both villages, mosquitoes were sampled in three habitat types: human settlements, plantations (including oil palm, rubber and fruit orchards) and forest edges, with two sample points for each habitat type in each village, totaling 12 sample locations. All sample points were at least 50 m from the next closest sampling point and positioned outside away from buildings and paths, except for at human settlements, where collections were made within 5 m of a dwelling. Monthly mosquito collections started in August 2015 and finished in November 2016. Collections were made for two successive nights at all sampling points in one village simultaneously, and then at the second village the following week, every month for 15 months data, except in October 2016 when data could not be collected, giving a total of 360 discrete catches over 60 collection nights.

Mosquitoes were sampled using the human landing catch (HLC) method, as this is still the most efficient method tested locally^16^, with only *Anopheles* species retained for further analysis. In HLCs, a volunteer collected mosquitoes by exposing their lower legs (from knee downwards) and collecting each mosquito that landed on them in a plastic specimen tube (2 cm diameter × 6 cm) with a piece of moist tissue at the bottom. Each HLC was performed by two persons who collected mosquitoes for six consecutive hours, from 18:00 to 00:00 hrs, thus covering the peak biting time of most *Anopheles* species^17^, with collectors rotated between each station. The HLC collectors were regularly and randomly monitored by a supervisor. The morning following collection, *Anopheles* mosquitoes were mounted and placed individually in a labeled Eppendorf tube, including hour of collection to record biting profile over the course of the collection period. Samples were then taken to the laboratory at Universiti Malaysia Sabah campus in Kota Kinabalu for further processing.

The HLC done by the volunteers were performed in accordance with relevant guidelines and regulations as approved by the Ethics committee.

### Identification of *Anopheles* species

*Anopheles* specimens were identified morphologically to species within 24 hours using published identification keys. For the *Leucosphyrus* group, the key of Sallum *et al*.^18^ was used and for other groups the keys developed by Rattanarithikul *et al*.^19^ were used. The identified specimens were kept individually in clean 1.5 mL microfuge tubes and stored at −20 °C until used for molecular analysis.

### Total DNA extraction and PCR of malaria parasites

Each *Anopheles* specimen was placed inside a clean 1.5 mL microfuge tube and the tissue was homogenized using a homogenizer. The total DNA was extracted from each specimen following the method of Phillips and Simon^20^ and stored at −30 °C until PCR analysis. Detection of malaria parasites was performed using the nested PCR *Plasmodium* genus-specific method described by Singh *et al*.^21^. When a sample was found positive for malaria parasites, a second nested PCR was performed to determine the *Plasmodium* species using species-specific primers in singleplex PCR^1,5,21,22^. Primers of nine species of *Plasmodium* were used in this study: *P*. *coatneyi*, *P*. *inui*, *P*. *fieldi*, *P*. *cynomolgi*, *P*. *knowlesi*, *P*. *falciparum*, *P*. *vivax*, *P*. *malariae* and *P*. *ovale*. All these species have been recorded in Malaysia, although *P*. *ovale* is an imported species.

Both first and second PCRs were performed with 25 μl final volume. The reaction components were prepared by mixing 5.0 μl of 5X PCR buffer, 0.5 μl of dNTPs (10 mM) mixture, 3.0 μl of 25 mM MgCl_2_, 1.0 μl each of 10 μM forward and reverse primers, 0.3 μl of Taq DNA polymerase (5 U/μl), 2.0 μl of DNA template and sterile dH_2_O up to 25 μl final volume. After the first PCR was completed, 2.0 μl of the first PCR product was used as a template in the second PCR. The PCR conditions used were: an initial denaturation at 95 °C for 5 min, followed by 35 cycles of denaturation at 94 °C for 1 min, annealing for 1 min, and extension at 72 °C for 1 min, and a final extension at 72 °C for 5 min. The annealing temperature was set based on the optimum temperature of the primers (Supplementary Table S2).

### Landscape classification and quantification

**Figure 2:**
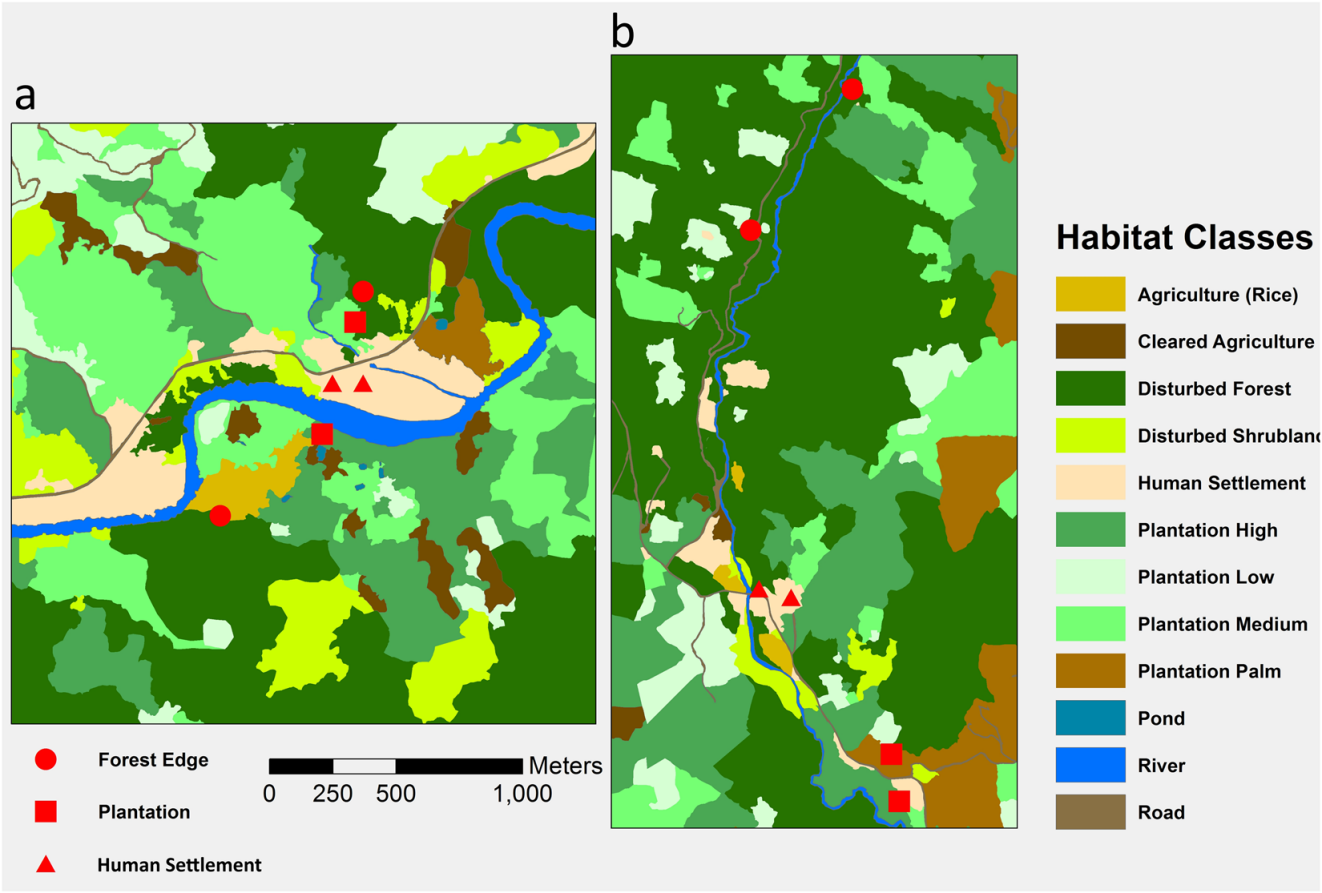
Land cover classifications in Ranau and Keningau. (**a**) Kampung Paus in Ranau district. (**b**) Kampung Keritan Ulu in Keningau Distrcit. Red markers indicate mosquito collection points at human settlements (triangles), plantations (squares) and forest edges (circles).

To determine the landscape characteristics of the study areas, very high-resolution satellite images were acquired from Bing Maps and Google Earth for areas covering the collection points in Kampung Paus (Ranau) and Kampung Keritan Ulu (Keningau). Imagery dates closest to the start of data collection were July 2014 for Ranau and April 2015 for Keningau. Polygons representing areas of uniform land cover type were manually digitized from the imagery by an experienced spatial information scientist (AC) using ArcGIS Pro v.10.4.1 software and class defined to emphasize variation in structure (Fig. 2a,b). Forest cover, denoted as disturbed forest, was observed to have varying degrees of human disturbance activity throughout both regions and, thus all forest cover is not considered primary forest. Disturbed shrublands were identified as areas of shorter and less dense vegetation than areas of disturbed forests. However, these areas could represent forest recovery post-disturbance. Human settlements were identified by the presence for buildings and cleared area surround these buildings not apparently in use for agriculture. Plantations were identified as having tree cover that is of apparent uniform tree types and lower canopy variation than disturbed forest, palm trees being the most apparent tree type upon visual interpretation. Plantation areas where verified from ground-based observation. Plantation density designations of low, medium or high were based on the density of canopy cover; low representing less than 30%, medium 30%–60%, and high >60%. These density designations also denote an assumed structural variation, which high plantations having the largest trees and medium and low designations with underdeveloped trees. Agriculture was apparent in satellite imagery and rice fields added from ground-based observation. Additional classes of Road, Pond, and River were clearly identifiable from visual interpretation.

Locations of mosquito collection points, recorded in the field by GPS, were added to the landcover maps and overlaid with a 500 m buffer, which was clipped from the map to define the habitat local to each collection point. Within each buffer, the cumulative area of disturbed forest, plantation and human settlement was calculated to determine percent landcover. Additionally, perimeter length of disturbed forest was determined to derive estimates of the forest edge:area ratio, indicating local forest fragmentation, where more fragmented forest (smaller, more isolated patches) have a larger forest edge:area ratio^23^.

### Mosquito diversity and statistical analysis

Analyses were performed using the R programming language for statistical analysis (version 3.2.2)^24^. Species diversity was investigated using four indices for disturbed forest, plantation and human settlement, based on all *Anopheles* collected. These were total species richness (*S*) and rarefied species richness *S*_*r*_, which accounts for the increased likelihood of sampling rare species in larger sample sizes and is the species richness normalized to the smallest sample size from an ecotype, Simpson’s Diversity Index (1 − *D*), Shannon-Weiner Diversity Index (*H*), and evenness (*E*). Diversity calculations were derived from R package “vegan”^25^.

The variation in *Anopheles* species abundance between different habitats and villages was analysed by negative binomial Generalized Linear Mixed Models (GLMMs). Habitat was set as a fixed effect and sampling night as a random effect. Models incorporating zero-inflation parameters were tested, but this was found to have a non-significant effect on model fit and was not included as a parameter in the model. Preliminary GLMM analysis showed that the negative binomial distributions gave better than, or similar, fits to the Poisson distribution. Tukey contrasts (α = 0.05) were used to compare differences in species abundance between habitats and villages.

To assess the effect of landcover on mosquito abundance, data were transformed to log(catch + 1) and analysed using a linear model, with habitat type (forest edge, plantation, human settlement) and forest edge:area ratio within a 500 m buffer of the collection point set as factors.

### Ethical clearance

This project was approved by the Ethics Committee of Universiti Malaysia Sabah (JKEtika 1/16(3)). All volunteers who carried out mosquito collections gave informed consent and were offered antimalarial prophylaxis throughout the study period.

## Results

### Composition of *Anopheles* species

A total of 1,071 *Anopheles* mosquitoes were collected, representing fourteen different species (Table 2). The total numbers of species caught in each village were similar (10 in Keningau and 11 in Ranau), although more individuals were caught at Ranau (620 versus 451 at Keningau). The five most abundant species in both villages were the same: *An. balabacensis*, *An. barbumbrosus*, *An. donaldi*, *An. maculatus* and *An. tessellatus*. However, the ranking of species abundance was different: in Keningau, *An. maculatus* (26.2%) was dominant, but it constituted only 2.4% of the total catch in Ranau. Conversely, in Ranau, *An. donaldi* was the dominant species (40.5%), whereas it accounted for 23% of *Anopheles* caught in Keningau. In both villages, the proportion of *An*. *balabacensis* was similar (23-25%) and more mosquitoes (45-49%) were caught in the forest edge than the other habitats.

**Table 2:** Total *Anopheles* species sampled at study sites in Ranau and Keningau for the period of August 2015 to November 2016.

*Anopheles* species	Ranau			Total	%	Keningau			Total	%
	HU	PL	FE			HU	PL	FE		
*An. argyropus*	0	0	0	0	0.0	0	1	0	1	0.2
An. balabacensis*	18	61	74	153	24.7	24	32	46	102	22.6
*An. barbumbrosus*	23	44	53	120	19.4	28	17	34	79	17.5
An. donaldi*	57	98	96	251	40.5	28	18	58	104	23.1
*An. vagus*	0	0	0	0	0.0	1	0	0	1	0.2
*An. kochi*	1	4	8	13	2.1	0	1	1	2	0.4
An. latens*	0	0	1	1	0.2	0	0	0	0	0.0
*An. maculatus*^a^	3	7	5	15	2.4	26	30	62	118	26.2
*An. montanus*	0	0	1	1	0.2	0	0	0	0	0.0
*An. paeditaeniatus*	0	1	0	1	0.2	0	0	0	0	0.0
*An. pujutensis*	0	0	1	1	0.2	0	0	0	0	0.0
An. sundaicus*	0	0	0	0	0.0	0	3	0	3	0.7
*An. tessellatus*	9	15	39	63	10.2	10	12	18	40	8.9
*An. umbrosus*	0	0	1	1	0.2	0	0	1	1	0.2
Total individuals	111	230	279	620		117	114	220	451	
%	17.9	37.1	45.0			25.9	25.3	48.8		
Total species	6	7	10	11		6	8	7	10	

HU = human settlement, PL = plantation, FE = forest edge. *Denotes species of medical importance.

### Species diversity

Anopheline species richness decreased from sites at forest edges, to plantations, to settlements, and even when adjusted to the lowest sample size (228 individuals in human settlements), rarefied richness was found to be lowest in human settlements (Table 3). Both Simpson’s and Shannon-Weiner diversity indices were highest at forest edges and lowest in human settlements (Table 3).

**Table 3:** Anopheline species diversity indices by habitat.

Species diversity index	HU	PL	FE
Species richness *S*	7	9	10
Rarefied species richness *S*_*r*_	5.3	5.9	5.9
Simpson’s Index 1 − *D*	0.75	0.76	0.79
Shannon-Weiner Index *H*	1.5	1.6	1.7
Evenness *E*	0.79	0.73	0.72

HU = human settlement; PL = plantation; FE = forest edge.

### Variation in catch with habitat

Landcover classifications for the two study villages are shown in Fig. 2. In Kampung Paus in Ranau (Fig. 2a), within the 500 m buffer surrounding collection sites, disturbed forest accounted for average landcover of 7% around human settlement sites, 12% around plantation sites and 22% around forest edge sites. The average percentage of disturbed forest was generally higher in Kampung Keritan Ulu in Keningau (Fig. 2b), with 30%, 37% and 67% around human settlement, plantation and forest edge sites, respectively. Plantation landcover varied less between the two villages. The buffers around human settlement, plantation and forest edge sites contained, respectively, 29%, 26% and 17% plantation in Ranau, and 31%, 20% and 11% plantation in Keningau. The percentage area attributed to human settlement was relatively small in both villages and across all sampling sites; in Ranau this ranged from 12% around forest edge sites, to 13% around human settlement and 14% around plantation, while in Keningau human settlement collection sites had an average of 7% of this landcover type, plantation only 2%, while forest edge sites had no land classified as human settlements in the surrounding 500 m.

**Figure 3:**
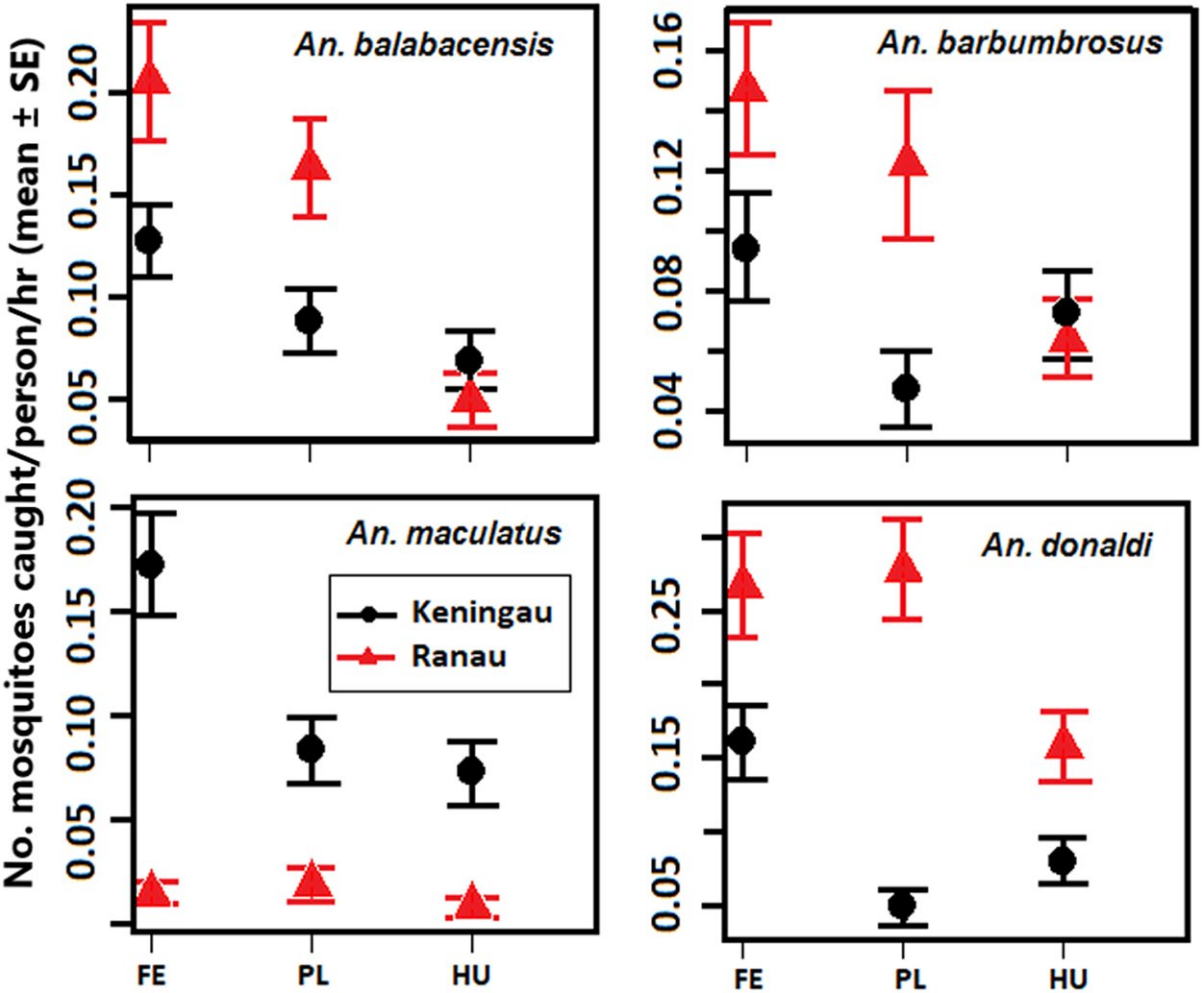
Variation in mosquito catch per person per hour (mean ± SE) according to habitat type for the four most common *Anopheles* species in Ranau and Keningau. HU = human settlement, PL = plantation, FE = forest edge.

Subsequent analysis focuses on the four most abundant species of medical importance identified in Table 2. The mean nightly catch of *An. balabacensis*, *An. donaldi, An. barbumbrosus* and *An. maculatus* in both villages decreased from forest edge, to plantation, to human settlement (Fig. 3). *An. maculatus* were present in very low numbers in Ranau and trends could not be observed.

Habitat type significantly affected predicted mean catch per night for the four medically important species detected (Table 4). In both study locations, *An. balabacensis* and *An. donaldi* were caught more frequently at forest edges than around human settlements. In Keningau, *An. maculatus* was also caught in higher numbers at forest edges, as was *An. barbumbrosus* in Ranau. However, the mean numbers of *An. barbumbrosus* in Keningau and *An. maculatus* in Ranau were not significantly different between the three habitats.

**Table 4:** Predicted mean nightly catch ± 95% SE of four mosquito species from different habitats in Keningau and Ranau.

Site	Species	GLMM-predicted means ± 95% SE			Significance level
		Human settlement	Plantation	Forest edge	
Keningau	*An. balabacensis*	0.0549 ± 0.0190a	0.0702 ± 0.0234ab	0.1010 ± 0.0242b	p < 0.05
	*An. donaldi*	0.0550 ± 0.0295a	0.0342 ± 0.0224a	0.1099 ± 0.0523b	p < 0.001
	*An. maculatus*	0.0394 ± 0.0177a	0.0454 ± 0.0195a	0.0936 ± 0.0336b	p < 0.001
	*An. barbumbrosus*	0.0557 ± 0.0219	0.0363 ± 0.0152	0.0740 ± 0.0209	no sig. dif.
Ranau	*An. balabacensis*	0.0232 ± 0.0109a	0.0762 ± 0.0326b	0.0955 ± 0.0373b	p < 0.01
	*An. donaldi*	0.1550 ± 0.0432a	0.2640 ± 0.0699ab	0.25579 ± 0.0516b	p < 0.001
	*An. maculatus*	0.00800 ± 0.0076	0.0185 ± 0.0127	0.0132 ± 0.0067	no sig. dif.
	*An. barbumbrosus*	0.0535 ± 0.0228a	0.0968 ± 0.0389ab	0.1194 ± 0.0393b	p < 0.05

Results from Generalized Linear Mixed Models with Tukey’s contrasts. Different letters along a row indicate a significant difference at the stated significance level.

**Figure 4:**
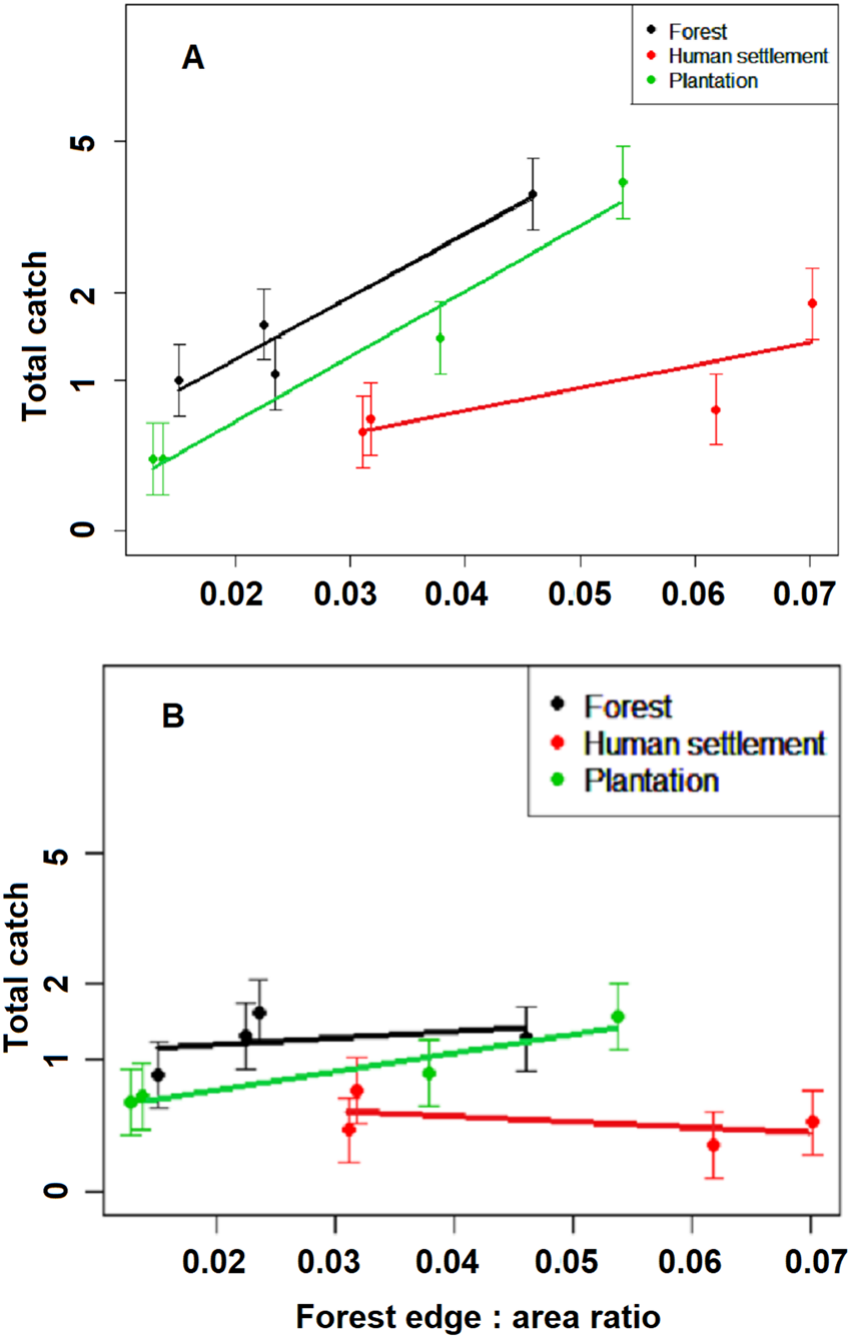
Total catch of *Anopheles* species (mean ± se) in three habitats in relation to the edge-area ratio of surrounding disturbed forest. Mean based on total sampling for 15 months at twelve collection points. Forest edge:area ratio calculated for a 500 m buffer surrounding each collection point. (**A**) *Anopheles donaldi*. (**B**) *Anopheles balabacensis*.

The relationship between catch and forest fragmentation was analyzed for *An. balabacensis* and *An. donaldi*, as these were the only two species found to be positive for *P. knowlesi* (see ‘Infection rate of *Anopheles* species’). Across all habitats, significantly more *An. donaldi* were caught at collection points with a greater forest edge:area ratio in the surrounding disturbed forest (Fig. 4A; F = 22.2; p < 0.001). This was not the case for *An. balabacensis*, where there was no significant difference in catch with respect to disturbed forest edge:area ratio in the 500 m buffer surrounding each collection point, irrespective of the habitat type (Fig. 4B; F = 1.7; p = 0.18).

### Temporal variation in catch

**Figure 5:**
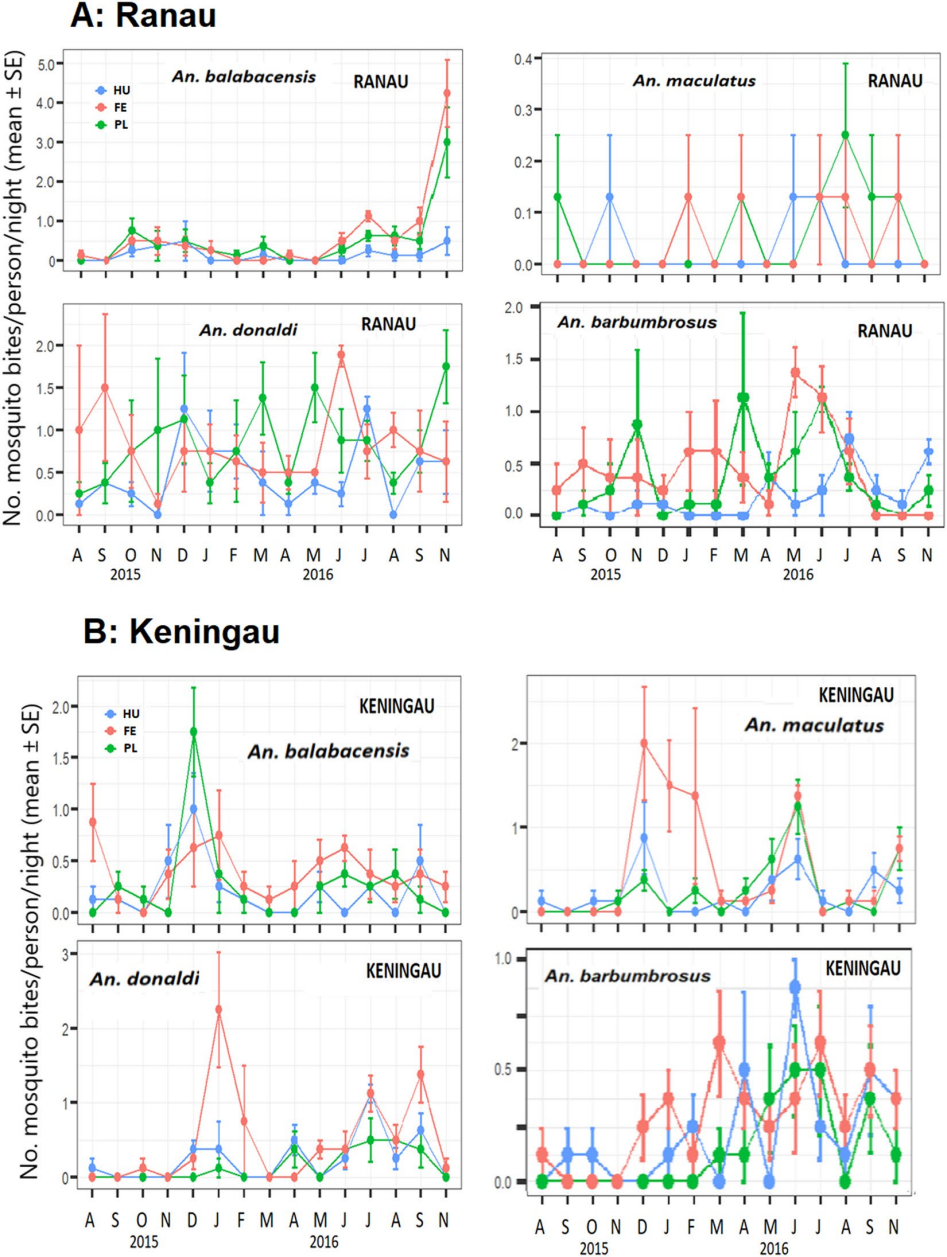
Number of mosquito bites/ person/ night (mean ± SE) across fifteen sampling months in Ranau and Keningau. HU = human settlement, PL = plantation, FE = forest edge. Sampling could not be done in October 2016. Note x-axis scales differ. (**A**) Kampung Paus in Ranau district. (**B**) Kampung Keritan Ulu in Keningau district.

Mosquito biting rates varied across the sampling period, showing seasonal peaks for most species, which were caught in greater numbers between November 2015 and January 2016, June and July 2016, and September and November 2016 (Fig. 5A,B). The number of *An. balabacensis* has a discernible peak in Ranau and Keningau, although the peak occurred in November 2016 in Ranau, but in December 2015 in Keningau. In Ranau, the trends for the other three species are not clear, whereas in Keningau, these species showed bimodal peaks in abundance between December 2015 and January 2016, and again between June and July 2016.

**Figure 6:**
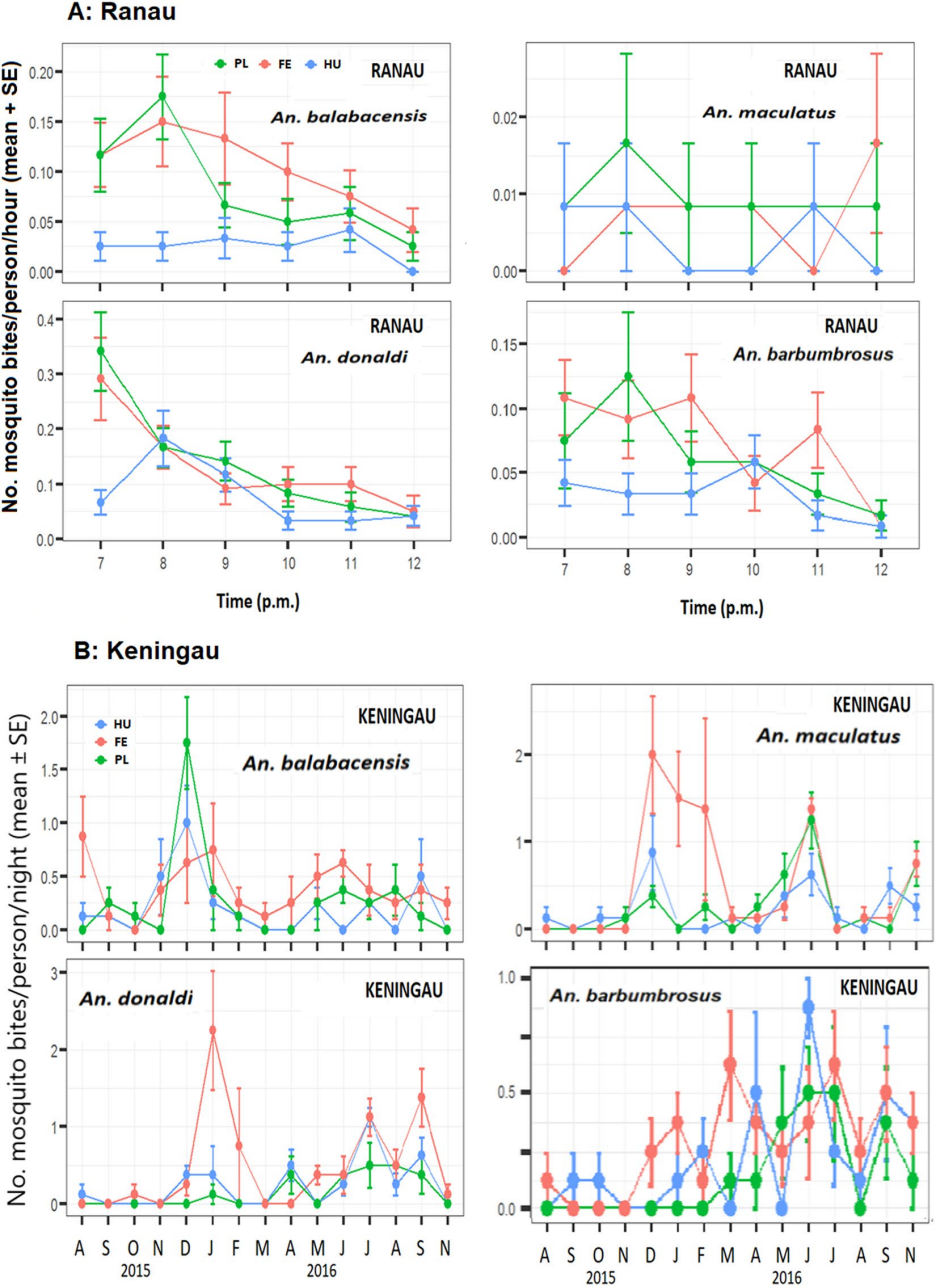
Biting times of *Anopheles* species (mean bites per person per hour ± SE) in three habitats of Keningau from 6 pm to midnight. HU = human settlement, PL = plantation, FE = forest edge. Mean based on total sampling for 15 months. Note x-axis scales differ. (**A**) Kampung Paus in Ranau district. (**B**) Kampung Keritan Ulu in Keningau district.

Biting time of *An. balabacensis* peaked at 7-8 p.m. in both Ranau and Keningau and decreased later, although *An. donaldi* and *An. maculatus* appeared to bite more at 6-7 p.m. (Fig. 6A,B). In general, the numbers of mosquitoes biting decreased later into the night. Peak biting time in settlements lagged those observed in the other two habitats.

### Infection rate of Anopheles species

A total of 1,069 individuals (99.8% of total catch) were assayed for *Plasmodium* infection using PCR. These were 253 *An. balabacensis*, 355 *An. donaldi*, 133 *An. maculatus*, 199 *An. barbumbrosus*, 103 *An. tesellatus* and 26 of the other rarer species (Supplementary Table S3). Positive results are presented in Table 5. Malaria parasites were only detected in *An. balabacensis* and *An. donaldi*. One *An. balabacensis* from Ranau was found to be positive with *P. knowlesi*. However, in Keningau, two *An. donaldi* tested positive for *P. knowlesi* parasites. Other simian malaria parasites detected were *P. cynomolgi* (in *An. balabacensis* and *An. donaldi*) and *P. inui* (in *An. balabacensis* only), with one unidentified *Plasmodium* species. Six out of seven positive specimens were collected from the forest edge habitat; no positive specimens were found in human settlements.

**Table 5:** *Anopheles* individuals positive for *Plasmodium* species.

Site	Habitat	Collection period	Collection time	*Anopheles* sp.	*Plasmodium* sp.
Ranau	PL	Nov 2016	8–9 pm	*An. balabacensis*	*P. knowlesi*
	FE	Oct 2015	7–8 pm	*An. balabacensis*	*P. cynomolgi*
	FE	Aug 2016	9–10 pm	*An. balabacensis*	*P. inui*
Keningau	FE	Jan 2016	6–7 pm	*An. donaldi*	*P. cynomolgi*
	FE	Sept 2016	9–10 pm	*An. donaldi*	*P. knowlesi*
	FE	Sept 2016	9–10 pm	*An. donaldi*	unknown
	FE	Sept 2016	6–7 pm	*An. donaldi*	*P. knowlesi*

PL = plantation, FE = forest edge.

## Discussion

Overall, the species composition of *Anopheles* was found to be similar between Kampung Paus in Ranau and Kampung Keritan Ulu in Keningau, even though these villages are located approximately 97 km from each other. Both localities share the same major species, namely *An. balabacensis*, *An. donaldi*, *An. maculatus* and *An. barbumbrosus*, and there is no single predominant species. This contrasts with previous results from Kudat district (Fig. 1), where the confirmed *P. knowlesi* vector *An. balabacensis* dominates, typically constituting 90-96% of *Anopheles* found in HLCs (Supplementary Table S4)^17^. Kudat is a low-lying area to the north of Sabah and sampling sites in this district tend to be at low elevations relative to Ranau and Keningau, typically around 19-180 m above sea level. Fornace *et al*.^11^ found associations between lower elevations and *P. knowlesi* incidence; in the present study Ranau and Keningau are both high elevation districts with a high number of *P. knowlesi* cases, but with a low proportion of *An. balabacensis* relative to other species in mosquito collections (Supplementary Table S1). Although we did not test this empirically, this could suggest that, alongside elevation, other factors may be locally important in mediating disease transmission.

The forest edge had the highest abundance, richness and diversity of mosquito species, while the human settlement the lowest. Old-growth and secondary forest, which occur around both study villages, offer a range of environmental niches suitable for diverse *Anopheles* species; herein refugia, suitable oviposition sites and other non-human hosts for many *Anopheles* species are plentiful^26,27^. It is possible that some of the mosquitoes caught in the settlements may be “vagrant” species flying in from the surrounding forests, as indicated by the apparent time lag of an hour or so in peak biting time for key species in human settlements compared to forest edge and plantations and this warrants spatially explicit investigation. Oil palm plantations are monocultures with minimal flora diversity^28^, although when established, they may provide anopheline breeding sites from wheel ruts and footpath depressions filled with water and potential refugia within the palms’ structure and their associated epiphytes^29^ which may explain the intermediate abundance of vectors between the three habitats. However, previous literature suggests this is unlikely, as no *Anopheles* were found resting in plantations in oil palm and rubber plantations in Kudat^30^ and malaria vector abundance was found to decrease in areas cultivated for oil palm^31^. More mosquitoes were trapped during the period between December and February, and May and July, which coincides with Sabah’s two main rainy seasons. The rain would provide more breeding grounds in the form of temporary rain pools, road puddles, puddles in rocks, animal hoof prints in the forests, plantations and at human settlements^32,33^. It has been shown in Côte d’Ivoire that monthly malaria incidence was positively associated with the quantity of rain one and two months before^34^. Moderate precipitation tends to increase potential breeding grounds although too much rainfall can flush away breeding habitats temporarily.

Prior to the current study, the only mosquito species found to be infected with *P. knowlesi* were members of the *Leucosphyrus* group^35^. The locally incriminated vector in Sabah is *An. balabacensis*, which falls within the *Leucosphyrus* complex (part of the *Leucosphyrus* group). In our study, we collected two *An. donaldi*, a member of the *Barbirostris* group, both of which were confirmed positive for *P. knowlesi* via PCR. Morphological vector species identification was independently verified by three experienced vector biologists, and PCR for *Plasmodium* detection was repeated five times. This finding is not sufficient to incriminate *An. donaldi* as a vector of *P. knowlesi* to humans, as we used whole mosquito samples for parasite detection, thus the positive samples could be carriers of *P. knowlesi*, rather than infectious vectors. As *An. donaldi* is a confirmed vector of human malaria parasites and positive specimens were collected via human landing catches, it does raise questions about the potential role of *An. donaldi* in zoonotic transmission. If subsequently incriminated through positive detection of parasites in the head or salivary glands, this would have implications for public health attempts to manage *P. knowlesi* in areas where *An. balabacensis* and *An. donaldi* coexist, as their bionomics differ and may require different intervention strategies, particularly as *An. donaldi* will enter dwellings to feed, highlighting the importance of maintaining bed net coverage.

It may also be the case that variations in response to environmental change vary both between and within species. For example, in Sarawak, in the four years immediately after conversion of a forest area to plantations of oil palm, *An. donaldi* showed a significant decline in abundance of 64%^31^, whereas work in the Kinabatangan area of Sabah reported exophagic *An. donaldi* had replaced *An. balabacensis* as the dominant vector of *P. falciparum* following deforestation and the implementation of malaria vector control activities^36^. In our study, although we did not observe a predominance of *An. donaldi*, we found a greater abundance of *An. donaldi* at collection points where the surrounding forest patches had a high degree of fragmentation, so the availability of edge habitats may be favourable to *An. donaldi*. With respect to *An. balabacensis*, we did not find evidence that forest fragmentation affected abundance. A recent study showed *An. balabacensis* preferred disturbed, logged forest over primary forest^37^. Together these results imply a degree of resilience to degraded forests, irrespective of edge availability or size of patch, although the mechanism underlying this would require further exploration. Precise landscape characteristics, and anthropogenic changes in the biotic and abiotic composition of habitats, may therefore be critical in determining how suitable the overall forest-matrix environment is to the success of these anopheline species.

We collected only one *An. balabacensis* that returned a positive result for *P. knowlesi* by PCR. Previous studies also showed that the number of *An. balabacensis* infected with *P. knowlesi* is extremely low, for example as low as 0.14% (2/1425)^38^ and often zero (out of 641^39^; out of 1,616^40^). The infected mosquitoes were mostly collected in the early night as reported previously^38,39^. It is very likely that most *An*. *balabacensis* are infected in the forest where the reservoir hosts abound. Host-seeking *An. balabacensis* are attracted to both humans and macaques^16^ and this specific trait in host-seeking behavior, coupled with increased potential for interaction between reservoir, vector and human host at forest edges^35^, may increase the likelihood of human clinical infection with *P*. *knowlesi*. Further research is required to establish whether *An. donaldi*, which we found to be carrying *P. knowlesi* parasites, plays an active role in *P. knowlesi* transmission. Other potential vectors in the study area include *An. maculatus*, a known vector of human malaria in peninsular Malaysia^41^. We also found specimens of both *An. balabacensis* and *An. donaldi* to be positive for *P. cynomolgi*, a *Plasmodium* species that was recently recorded in the first naturally-acquired human infection^5^; *An. donaldi* has also been shown to be a vector of an unidentified *Plasmodium* species^36^. Thus, public health surveillance should be alert to the presence of vectors carrying a range of malaria parasites and the possibility that *P. cynomolgi* may become a zoonosis causing routine human infection.

The frontline vector control methods practiced in Malaysia under the malaria elimination programme for human-specific malaria, i.e. the application of insecticides in houses either through use of long-lasting insecticide treated nets or indoor residual spraying with K-Othrine, appear to be less effective against *P*. *knowlesi* as its confirmed vector bites outdoors where these interventions are not effective. If *An. donaldi* is a vector, presumably it would continue to be controlled by these standard interventions, although we note in the current study this species was readily caught biting outdoors. *Plasmodium knowlesi* infection risk therefore appears to be linked to evening exposure to vectors in peri-domestic or agricultural settings^40^. Given the limited impact of current vector control on zoonotic vectors, control of *P. knowlesi* in endemic areas requires more information on the vector bionomics within a range of habitats that reflect current land use patterns alongside data on human movement patterns. Data on differences in malaria vector abundance and ecology are critical for understanding the potential to reduce zoonotic transmission both locally within the current *P. knowlesi* epicentre in Sabah, and other South East Asian regions where this disease is present and other parasitic vector-borne diseases may be emerging. Future research might be directed towards elucidating the potential role of *An. donaldi* in *P. knowlesi* transmission and the mechanisms underlying the paradox of sustained transmission in areas where vectors such as *An. balabacensis* have a low infection rate by *P. knowlesi* and are relatively small in population size.

## Supplementary information

Vector compositions change across forested to deforested ecotones in emerging areas of zoonotic malaria transmission in Malaysia

## Data Availability

All primary data is presented within the manuscript. Data sets can be made available after a written expression of request for data with no apparent competing interest and in compliance with the project’s data sharing agreements.
