# Author Correction: Vector compositions change across forested to deforested ecotones in emerging areas of zoonotic malaria transmission in Malaysia

**DOI:** 10.1038/s41598-019-53744-8

**Published:** 2019-11-20

**Authors:** Frances M. Hawkes, Benny O. Manin, Amanda Cooper, Sylvia Daim, Homathevi R., Jenarun Jelip, Tanrang Husin, Tock H. Chua

**Affiliations:** 1 Natural Resources Institute, University of Greenwich at Medway, Chatham Maritime, Kent, ME4 4TB UK; 2 Universiti Malaysia Sabah, Kota Kinabalu, Sabah 88400 Malaysia; 3 Royal Botanic Gardens Kew, Richmond, Surrey, TW9 3AE UK; 4 Disease Control Division, Ministry of Health, Federal Government Administration Centre, Putrajaya, Malaysia; 5 Division of Public Health, Sabah Department of Health, Kota Kinabalu, Sabah Malaysia

Correction to: *Scientific Reports* 10.1038/s41598-019-49842-2, published online 16 September 2019

In this Article, Figure 6B is a duplication of Figure 5B. The correct Figure 6 appears below as Figure 1.

**Figure 1:**
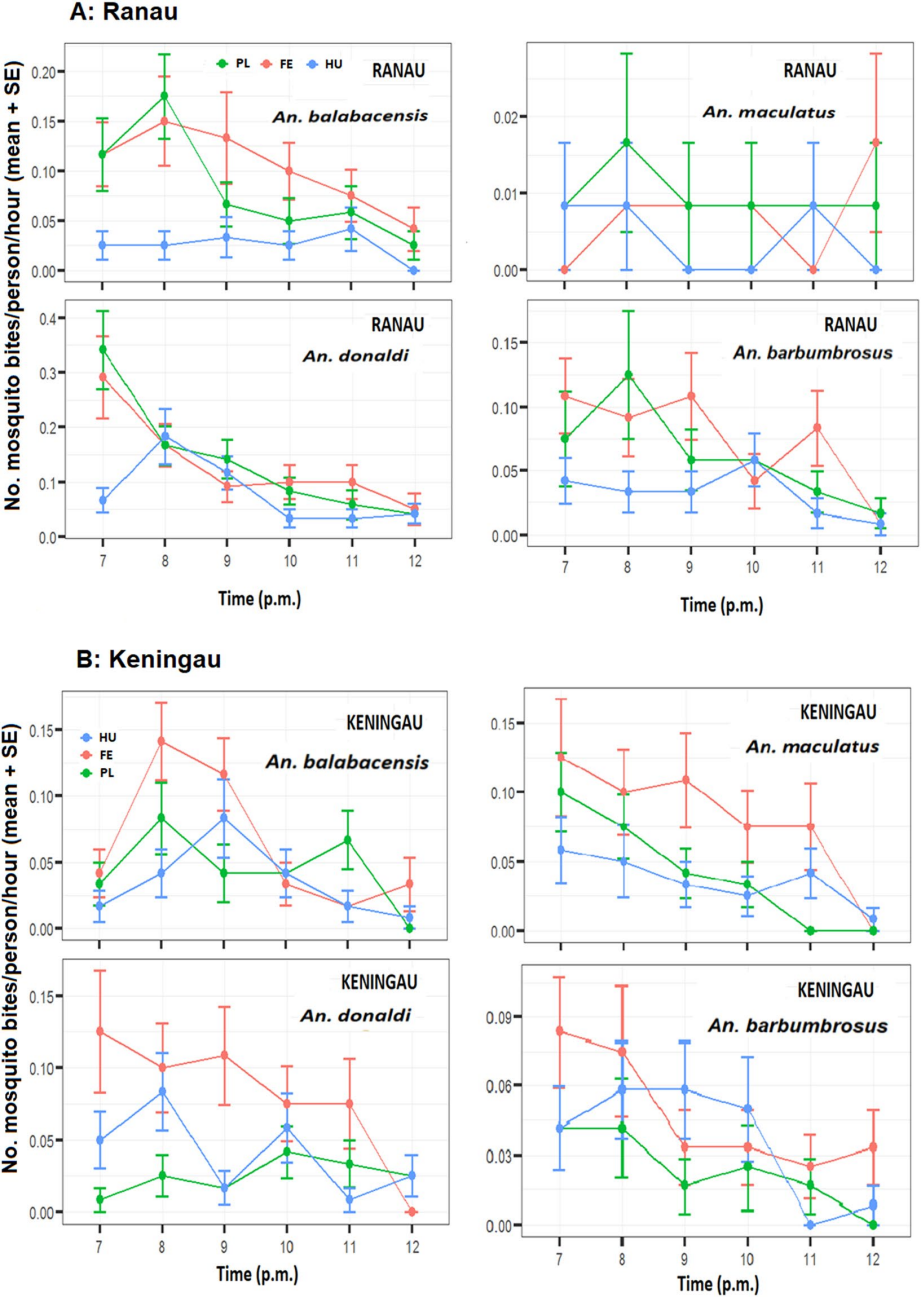
.

