# Supplementary material for: Vector compositions change across forested to deforested ecotones in emerging areas of zoonotic malaria transmission in Malaysia
Source: Sci Rep. 2019 Sep 16;9:13312. doi: 10.1038/s41598-019-49842-2 (PMC6746737; doi:10.1038/s41598-019-49842-2)
Supplement: Supplementary file 1 — Vector compositions change across forested to deforested ecotones in emerging areas of zoonotic malaria transmission in Malaysia [file 41598_2019_49842_MOESM1_ESM.pdf]

SUPPLEMENTARY INFORMATION

Vector compositions change across forested to deforested ecotones in emerging areas of zoonotic malaria transmission in Malaysia

Frances M. Hawkes, Benny O. Manin, Amanda Cooper, Sylvia Daim, Rahman Homathevi, Jenarun Jelip, Tanrang Husin, and Tock H. Chua*

S1 Table: *Plasmodium knowlesi* cases in Kudat, Ranau and Keningau districts for the years 2011 - 2016. Figures in brackets are each district's cases as a percentage of the total number of all *Plasmodium* cases for that year.

District	2011	2012	2013	2014	2015	2016	Total
Ranau	77 (40%)	124 (50%)	172 (53%)	166 (40%)	116 (44%)	76 (46%)	731
Keningau	4 (2%)	33 (13%)	88 (27%)	169 (41%)	92 (35%)	78 (47%)	464
Kudat	111 (58%)	91 (37%)	66 (20%)	82 (20%)	53 (20%)	13 (8%)	416
Total	192	248	326	417	261	167	1611

S2 Table: Details of PCR primers used in PCR reactions for detection of *Plasmodium* parasites in *Anopheles* specimens.

Target	Genus/species	Primer set for PCR	Set primer	Primer name	Reference	Sequence (5' - 3')	Annealing tem. (^{o}C)	Size of PCR product (bp)
SSU-rRNA	*Plasmodium* genus	Sets 1 + 2	Set 1	rPLU1	1	TCAAAGATTAAGCCATGCAAGTGA	55	1640
				rPLU5	1	CCTGTTGTTGCCTTAAACTCC		
			Set 2	rPLU3	1	TTTTTATAAGGATAACTACGGAAAAGCTGT	62	240
				rPLU4	1	TACCCGTCATAGCCATGTTAGGCCAATACC		
	P. coatneyi	Sets 1 + 3	Set 3	PctF1	2	CGCTTTTAGCTTAAATCCACATAACAGAC	62	504
				PctR1	2	GAGTCCTAACCCCGAAGGGAAAGG		
	P. inui	Sets 1 + 4	Set 4	PinF2	2	CGTATCGACTTTGTGGCATTTTTCTAC	60	479
				INAR3	2	GCAATCTAAGAGTTTTAACTCCTC		
	P. fieldi	Sets 1 + 5	Set 5	PfldF1	2	GGTCTTTTTTTTGCTTCGGTAATTA	66	421
				PfldR2	2	AGGCACTGAAGGAAGCAATCTAAGAGTTTC		
	P. cynomolgi	Sets 1 + 6	Set 6	CY2F	2	GATTTGCTAAATTGCGGTCG	60	137
				CY4R	2	CGGTATGATAAGCCAGGGAAGT		
	P. knowlesi	Sets 1 + 7	Set 7	PkF1140	3	GATTCATCTATTAAAAATTTGCTTC	50	424
				PkR1550	3	GAGTTCTAATCTCCGGAGAGAAAAGA		
	P. falciparum	Sets 1 + 8	Set 8	NewPLFshort	4	CTATCAGCTTTTGATGTTAG	53	370
				FARshort	4	GTTCCCCTAGAATAGTTACA		
	P. vivax	Sets 1 + 9	Set 9	NewPLFshort	4	CTATCAGCTTTTGATGTTAG	53	476
				VIRshort	4	AAGGACTTCCAAGCC		
	P. malariae	Sets 1 + 10	Set 10	NewPLFshort	4	CTATCAGCTTTTGATGTTAG	53	241
				MARshort	4	TCCAATTGCCTTCTG		
	P. ovale	Sets 1 + 11	Set 11	NewPLFshort	4	CTATCAGCTTTTGATGTTAG	53	407
				OVRshort	4	AGGAATGCAAAGARCAG		

1. Singh, B. *et al.* A genus- and species-specific nested polymerase chain reaction malaria detection assay for epidemiologic studies. Am. J. Trop. Med. Hyg. **60,** 687-692 (1999).

2. Imwong, M. *et al.* Spurious amplification of a *Plasmodium vivax* small-subunit RNA gene by use of primers currently used to detect *P. knowlesi*. J. Clin. Microbiol. **47,** 4173-4175 (2009).

3. Lee, K. S. *et al. Plasmodium knowlesi*: reservoir hosts and tracking the emergence in humans and macaques. PLoS Pathog. 7, e1002015 (2011).

4. Ta, T. H. *et al*. First case of a naturally acquired human infection with *Plasmodium cynomolgi*. Malaria J. **13**, 68 (2014).

S3 Table: Number of *Anopheles* individuals (total =1069) which were examined for *Plasmodium* infection. HU = Human settlement, PL = Plantation, FE = Forest edge.

Site	Eco-type	*An. argyropus*	*An. balabacensis*	*An. barbumbrosus*	*An. donaldi*	*An. vagus*	*An. kochi*	*An. latens*	*An. maculatus*	*An. montanus*	*An. paeditaeniatus*	*An. pujutensis*	*An. sundaicus*	*An. tessellatus*	*An. umbrosus*	Total
Ranau (n=620)	HU	0	18	23	57	0	1	0	3	0	0	0	0	9	0	111
	PL	0	61	44	98	0	4	0	7	0	1	0	0	15	0	230
	FE	0	74	53	96	0	8	1	5	1	0	1	0	39	1	279
	Tot	0	153	120	251	0	13	1	15	1	1	1	0	63	1	620
Keningau (n=449)	HU	0	25	26	29	1	0	0	26	0	0	0	0	10	0	117
	PL	1	32	17	18	0	1	0	30	0	0	0	3	12	0	114
	FE	0	44	34	58	0	1	0	62	0	0	0	0	18	1	218
	Tot	1	101	77	105	1	2	0	118	0	0	0	3	40	1	449
Total (Ranau + Keningau)		1	253	199	355	1	15	1	133	1	1	1	3	103	2	1069

S4 Table: Percentage of various *Anopheles* caught by HLC in Sabah in various recent studies. Ranau is administered under West Coast Division, Keningau in Interior Division, while Banggi Island, Paradason and Kudat are under Kudat Division.

Sampling period	August 2015 - November 2016 (this study)		August 2013 - July 2014 (1)		February 2014 - July 2014 (2)	November 2013 - January 2014 (3)
Site	Ranau	Keningau	Banggi Island	Paradason	28 locations in Kudat	Tajau Laut
Elevation (m above sea level)	223	509	24 - 76	24	19 - 180	29
Total individuals caught	620	451	1001	883	793	403
Number of species	11	10	8	6	12	10
An. aconitus	-	-	1.1	-	-	-
An. argyropus	-	0.2	-	-	-	-
An. baezai	-	-	-	-	0.1	-
An. balabacensis Baisas	24.7	22.6	94.2	96.0	80.8	40.2
An. barbirostris	-	-	0.2	1.2	-	-
An. barbumbrosus	19.4	17.5	-	-	4.7	1.5
An. donaldi Reid	40.5	23.1	1.3	1.4	4.3	0.7
An. flavirostris	-	-	0.5	-	-	-
An. indefinitus Ludlow	-	-	-	-	0.3	0.5
An. kochi Donitz	2.1	0.4	-	-	0.1	0.2
An. latens	0.2	-	-	-	-	4.7
An. maculatus Theobald	2.4	26.2	-	0.9	6.2	3.5
An. montanus	0.2	-	-	-	-	-
An. nigerrimus	-	-	-	-	-	-
An. peditaeniatus	0.2	-	-	-	0.4	0.2
An. pujutensis	0.2	-	-	-	-	-
An. separatus	-	-	-	-	-	-
An. sundaicus	-	0.7	-	-	-	-
An. subpictus	-	-	-	-	1.5	4.0
An. tessellatus Theobald	10.2	8.9	-	0.2	1.0	44.4
An. umbrosus	0.2	0.2	0.7	-	0.3	-
An. vagus	-	0.2	1.9	-	-	-
An. watsonii	-	-	0.1	0.2	-	-
An. whartoni	-	-	-	-	0.4	-

1. Wong, M. L. *et al.* Seasonal and spatial dynamics of the primary vector of *Plasmodium knowlesi* within a major transmission focus in Sabah, Malaysia. PLoS Negl. Trop. Dis. **9,** e0004135 (2015).

2. Manin, B.O. *et al.* Investigating the contribution of peri-domestic transmission to risk of zoonotic malaria infection in humans. PLoS Negl. Trop. Dis. **10,** e0005064 (2016).

3. Hawkes, F. *et al.* Evaluation of electric nets as means to sample mosquito vectors host-seeking on humans and primates. Parasites Vectors. **10**, 338 (2017).
